# Species differential regulation of COX2 can be described by an NFκB-dependent logic AND gate

**DOI:** 10.1007/s00018-015-1850-1

**Published:** 2015-02-20

**Authors:** Lan K. Nguyen, Miguel A. S. Cavadas, Boris N. Kholodenko, Till D. Frank, Alex Cheong

**Affiliations:** 1 Systems Biology Ireland, University College Dublin, Dublin 4, Ireland; 2 Instituto Gulbenkian de Ciência, Oeiras, Portugal; 3 Center for the Ecological Study of Perception and Action, University of Connecticut, Storrs, CT 06269 USA; 4 School of Life and Health Sciences, Aston University, Birmingham, B4 7ET UK

**Keywords:** NFκB signaling, COX2, Switch-like gene regulation, Logic AND gate, Kinetic modeling

## Abstract

Cyclooxygenase 2 (COX2), a key regulatory enzyme of the prostaglandin/eicosanoid pathway, is an important target for anti-inflammatory therapy. It is highly induced by pro-inflammatory cytokines in a Nuclear factor kappa B (NFκB)-dependent manner. However, the mechanisms determining the amplitude and dynamics of this important pro-inflammatory event are poorly understood. Furthermore, there is significant difference between human and mouse COX2 expression in response to the inflammatory stimulus tumor necrosis factor alpha (TNFα). Here, we report the presence of a molecular logic AND gate composed of two NFκB response elements (NREs) which controls the expression of human COX2 in a switch-like manner. Combining quantitative kinetic modeling and thermostatistical analysis followed by experimental validation in iterative cycles, we show that the human COX2 expression machinery regulated by NFκB displays features of a logic AND gate. We propose that this provides a digital, noise-filtering mechanism for a tighter control of expression in response to TNFα, such that a threshold level of NFκB activation is required before the promoter becomes active and initiates transcription. This NFκB-regulated AND gate is absent in the mouse COX2 promoter, most likely contributing to its differential graded response in promoter activity and protein expression to TNFα. Our data suggest that the NFκB-regulated AND gate acts as a novel mechanism for controlling the expression of human COX2 to TNFα, and its absence in the mouse COX2 provides the foundation for further studies on understanding species-specific differential gene regulation.

**Electronic supplementary material:**

The online version of this article (doi:10.1007/s00018-015-1850-1) contains supplementary material, which is available to authorized users.

## Introduction

Inflammation is a part of the immune system’s response to infection and injury. The usual outcome of an inflammatory cellular response is its successful resolution and repair of tissue damage, whereas persistence and dysfunction of the inflammatory response has been implicated in the pathogenesis of diseases such as arthritis, cancer, neurodegenerative and cardiovascular diseases [1].

Key players in the generation of the inflammatory response are the cyclooxygenase enzymes COX1 and COX2, which catalyze the conversion of arachidonic acid into pro-inflammatory prostaglandins and trigger the production of other pro-inflammatory chemokines and cytokines [2, 3]. COX1 is constitutively expressed in most tissues and is involved in cellular housekeeping functions [4], while the inducible isoform COX2 is expressed in response to inflammatory stimuli such as TNFα [5]. Consequently, a therapeutic strategy for inflammatory diseases has involved the inhibition of COX, specifically COX2 [3] (although adverse side effects have led to the withdrawal of selective COX2 inhibitors [6]). Anti-inflammatory candidate drugs are usually developed and tested in mouse models but have very poor success rates when moved to clinical trials [7]. A recent review of mouse models of inflammatory diseases has found no correlation between the responses in mouse models and human diseases [7]. Thus, COX2 inhibitors, which work well in mouse, can be unfavorable in humans.

Our work has focused on understanding the mechanisms regulating induced COX2 expression at the level of gene transcription. COX2 is regulated by a diverse group of transcription factors such as AP1, CRE, HIF, SP1 and STAT [8–11], but most importantly it is highly induced by pro-inflammatory stimulus and activated by NFκB [12]. Indeed, the human COX2 promoter was shown to contain two NFκB response elements (NREs) bound by the canonical NFκB subunits p50 and p65 [11, 13–15]. In the mouse COX2 promoter, only 1 NRE has been identified [16], suggesting a different regulation. The mechanisms determining the amplitude and dynamics of the NFκB-mediated regulation of COX2 expression are poorly understood. Given that NFκB is involved in the transcription of a wide variety of genes [17–19], some of which containing several NREs in their promoters, a key question has been how it is involved in the specific activation of these genes and the control of their activation amplitudes and dynamics. TNFα was shown to induce p65 oscillations [20, 21] in a concentration-dependent manner [22] and a genome-wide analysis of NFκB binding sites in the human genome showed that clusters of NREs increased gene transcription in response to an increasing gradient of NFκB concentration. This analog transcriptional response is predicted to become switch-like or digital when the binding cooperativity among the NREs increases [23].

In this study, we used a combination of quantitative kinetic modeling and experimental validation to describe a novel mechanism for controlling the expression of human cyclooxygenase 2 (hCOX2): a molecular logic AND gate composed of two NREs acting in tandem to give a switch-like property to hCOX2 expression. We also report that this logic gate is absent in the mouse COX2 promoter, which instead produced an analog response to NFκB. Thus, our results demonstrate an additional level of complexity in the specific gene activation by NFκB in different species.

## Materials and methods

### Cell culture

Human HEK293 and HT29 and mouse MEF cells were cultured in DMEM high-glucose medium supplemented with 10 % FCS and 100 U/ml penicillin–streptomycin in a 5 % CO_2_ humidified incubator.

### Western blot analysis

To prepare whole cell extracts, cells were lysed with RIPA buffer, centrifuged (14,000 rpm, 15 min, 4 °C) and the supernatant stored at −20 °C. For the preparation of nuclear and cytoplasmic extracts, cells were lysed in buffer A (10 mM HEPES pH 8, 1.5 mM MgCl_2_, 10 mM KCl, 200 mM sucrose, 0.5 mM DTT, 0.25 % NP-40 and 1× PIC), centrifuged (12,000 rpm, 1 min, 4 °C), the supernatant containing the cytosolic extract was stored at −20 °C. The nuclear pellet was washed again in buffer A, suspended in buffer C (20 mM HEPES pH 8, 420 mM NaCl, 0.2 mM EDTA, 1.5 mM MgCl_2_, 0.5 mM DTT, 25 % glycerol), incubated for 30 min on ice and pelleted again, the supernatant containing the soluble nuclear proteins was stored at −80 °C. Protein concentrations were quantified using a Lowry assay and normalized accordingly. Samples were separated by SDS-PAGE and immunoblotted using the following antibodies: goat COX2 (1:1,000, Santa Cruz, sc-1745), mouse β-actin (1:10,000; Sigma, A5441), rabbit p65 (1:1,000, Santa Cruz, sc-372), rabbit lamin A/C (1:1,000; Cell Signaling, 2032). Secondary antibodies, anti-mouse IgG HRP conjugate (1:1,000, Promega) and anti-rabbit IgG HRP conjugate (1:1,000, Promega) were used. Development was performed with the Detection kit Pierce^®^ ECL Western Blotting Substrate (Thermo Scientific). Stripping buffer was used to allow for the detection of all the above-mentioned antigens on the same membrane.

### Chromatin immunoprecipitation

Chromatin immunoprecipitation (ChIP) assays were performed as previously described with some modifications [15]. HEK293 and MEF cells fully confluent on T175 flasks were conditioned to 10 ng/mL TNFα for 45 min. Cells were fixed with 1 % formaldehyde in 10 mL fresh media for 10 min with agitation. Fixation was stopped with 125 mM glycine treatment for 5 min. Cells were scrapped with 1 mM PMSF in PBS, pelleted and resuspended in 1 mL ChIP buffer A (100 mM Tris pH 9.4, 10 mM DTT, PIC and 1 mM PMSF), after 10 min incubation with agitation cells were pelleted and resuspended in ChIP buffer D (10 mM Tris pH 8.0, 1 mM EDTA, 0.5 % empigen, PIC and 1 mM PMSF), and incubated on ice for 30 min. Samples were subjected to sonication (Bioruptor), cell debris was pelleted (14,000 rpm, 5 min, RT) and 400 µL of the supernatant containing the shred chromatin with an average size of 500–1,000 bp was mixed with 900 μL of ChIP dilution buffer (20 mM Tris pH 8, 150 mM NaCl, 2 mM EDTA, 1 % Triton, 1 mM PMSF) and pre-cleared with 50 µL of protein A/salmon sperm beads (16–157, Millipore) previously blocked with BSA in ChIP dilution buffer, and incubated overnight with agitation at 4 °C. Beads were centrifuged and the chromatin was quantified using a NanoDrop (Thermo Scientific), 60 µg of chromatin was diluted in ChIP dilution buffer to a final volume of 650 µL, 10 µL was taken as input, 1 µg of p65 antibody (rabbit, Santa Cruz, sc-372) or IgG control antibody (rabbit, Millipore, PP64B) was added and incubated overnight at 4 °C with agitation. This was followed by centrifugation and the supernatant from the spin step containing the chromatin:p65 immuno-complexes was incubated with 50 µL of protein A/salmon sperm beads (previously blocked with BSA in ChIP dilution buffer) for 2 h at 4 °C with agitation. Beads were washed twice with RIPA buffer (50 mM HEPES pH 7.8, 140 mM NaCl, 1 % Triton X-100, 1 mM EDTA, 0.1 % sodium deoxycholate and 0.1 % SDS) with 15-min incubation periods at 4 °C with agitation. This was followed by washing steps with high salt RIPA (RIPA supplemented with 350 mM NaCl), LiCl buffer (20 mM Tris pH 8.0, 250 mM LiCl, 0.5 % IGEPAL CA-360, 1 mM EDTA, 0.5 % sodium deoxycholate and 0.5 mM PMSF) and finally twice with TE buffer (10 mM Tris, 1 mM EDTA, pH 7.4). Immunocomplex elution was performed by adding 250 µL of elution buffer (1 % SDS, 0.1 M NaHCO_3_) to the centrifuged beads and heated with shaking at 65 °C. 240 µL of the supernatant was recovered and this step was repeated. 470 µL of elution buffer was now added to the input samples, and treated as the ChIP samples from now on. Samples were treated with RNAse A for 30 min at 37 °C. Crosslinks were reversed by incubating the samples overnight at 65 °C. Samples were than treated with proteinase K (42 °C, 2 h). The DNA was than isolated by phenol/chloroform precipitation, and resuspended in 60 µL nuclease free water. Purified DNA (3 µL) was amplified using human COX-2 promoter primers (NRE1: forward 5′-GGCAAAGACTGCGAAGAAGA-3′, reverse 5′-AAAATCGGAAACCCAGGAAG-3′; NRE2: forward 5′-CCTCGACCCTCTAAAGACGTA-3′, reverse 5′-AGCCAGTTCTGGACTGATCG-3′) using a thermocycler program (94 °C for 3 min; then 36 cycles of 94 °C for 20 s, 60 °C for 30 s, and 72 °C for 30 s; then a hold cycle of 10 °C). Samples were run on a 2 % agarose gel using ethidium bromide to visualize a 150-bp product for NRE1 and a 165-bp product for NRE2. For the mouse NRE site, we had technical difficulties in amplifying a single PCR product (data not shown), thus we used Taqman chemistry, which combines specific primers and probes to prevent the formation of unspecific products and primer dimers. Thus, chromatin precipitation analysis of the mouse NRE site was performed using quantitative-reverse-transcription PCR (qPCR), and the results are shown as fold change to the unstimulated p65 precipitated chromatin, after normalization to the input samples. The sequences for the Taqman reagents are: forward 5′-AGACTGCGCCCCAGT-3′, reverse 5′-CCGGGATCTAAGGTCCTAACTAAGG-3′, probe 5′-GGGAGAGGTGAGGGGAT-3′). The following program was used 50 °C for 2 min, followed by 95 °C for 10 min; then 39 cycles of 95 °C for 15 s followed by 60 °C for 60 s, in the 7900HT Fast Real-Time PCR System (Life Technologies).

### Transient transfections and Gaussia luciferase assay

All transfections were performed using Lipofectamine 2000 (Invitrogen) according to the manufacturer’s protocol. The empty vector used for cloning is the pGluc vector (NEB) driven by a minimal promoter [11]. The pGluc-COX2 Gaussia luciferase vector contains the sequence −4 to −631 of the human COX2 gene which includes two NFκB response elements (NREs) [11]. The pGluc-mCOX2 vector was made by inserting the sequence −441 to −116 of the mouse COX2 gene which includes one NFκB response element [16]. The pGluc-IL6 Gaussia luciferase vector was made by inserting the sequence +1 to −225 of the human interleukin-6 gene which includes one NFκB response element only [24]. The pGluc-2xIL6 Gaussia luciferase vector was made by inserting two copies of the sequence +1 to −225 of the human IL6 promoter to create an artificial OR gate. The sequence of the inserts used is provided in Supplementary Figures S1, S2, S5 and S6. Mutations to remove the NREs were introduced by site-directed mutagenesis. Concentration of plasmids used for transfection was 100 ng/100,000 cells unless otherwise stated. For the promoter concentration experiments, to maintain equal concentration of DNA transfected, we have used the empty vector plasmid DNA to buffer the transfection mix. Gaussia luciferase activity was measured using the Biolux Gaussia luciferase Flex Assay kit (NEB) in a plate reader (Synergy HT, Biotek).

### Analysis of transcriptional activity

A mathematical expression for the transcriptional activity *r* as a function of the TF concentration can be derived using a thermostatistical approach. It is useful to rescale the concentration of the relevant TF with its dissociation constant. Thus, a dimensionless, relative concentration variable [TF]_rel_ is obtained [25]. For the 1-site model and for the AND gate, the transcriptional activity as a function of [TF]_rel_ reads as [26].

$$r\left( {\left[ {\text{TF}} \right]_{\text{rel}} } \right) = r_{0} \frac{{1 + A_{\text{sat}} \gamma [{\text{TF}}]_{\text{rel}}^{n} }}{{1 + \gamma [{\text{TF}}]_{\text{rel}}^{n} }}$$

with exponents *n* = 1 for the 1-site model and *n* = 2 for the AND gate. Here, *r*_0_ is the baseline activity for zero TF concentration. The transcriptional activities *r*(*X*) and r_0_ are measured in arbitrary units. The parameters *γ* and *A*_sat_ are lump parameters that are composed of certain chemical binding energies [25, 26]. The parameter *A*_sat_ has a simple interpretation. The product *r*_0_·*A*_sat_ is the saturation transcriptional activity. The predicted fold change can be computed as well. To this end, the formula is divided by the baseline activity.

$${\text{Fold change}} = \frac{{1 + A_{\text{sat}} \gamma [{\text{TF}}]_{\text{rel}}^{n} }}{{1 + \gamma [{\text{TF}}]_{\text{rel}}^{n} }}$$

We see that *A*_sat_ corresponds to the fold change for saturation. Furthermore, the expression for the transcriptional activity can be cast into the form of a Hill function like

$$r\left( {\left[ {\text{TF}} \right]_{\text{rel}} } \right) = \beta + R_{\hbox{max} } \frac{{[{\text{TF}}]_{\text{rel}}^{n} }}{{d + [{\text{TF}}]_{\text{rel}}^{n} }}$$

with *r*_0_ = *β*, *γ* = 1/*d*, and *A*_sat_ = (*R*_max_ + *β*)/*β.* Although the relationship between the stimulus *X* (e.g., TNFα) and the relative transcription factor concentration [TF]_rel_ has to be modeled separately (e.g., by means of a linear or nonlinear mapping *X* → [TF]_rel_), the fact that in the thermostatistical model the exponent *n* is higher for the AND gate than for the 1-site model suggests that the AND gate exhibits more switch-like behavior relative to the promoter with a single binding site. In doing so, the analytical thermostatistical approach is consistent and complementary to the numerical kinetic modeling approach described below.

### Quantitative kinetic modeling of promoter activity mediated by transcriptional factors

To quantitatively analyze and predict steady-state dynamics of the COX2 gene expression under different modes of regulation by a transcriptional factor (e.g., NFκB), we developed three general kinetic models that describe: (1) a promoter that is regulated by a transcriptional factor through a single TF-Promoter binding site; (2) a promoter regulated by a transcriptional factor through two TF-Promoter binding sites following a OR gate and (3) a promoter regulated by a transcriptional factor through two TF-Promoter binding sites following an AND gate regulation. For convenience, we will refer to these models as the “1-site”, “2-site OR-gate” and “2-site AND-gate” models, respectively. These models are schematically illustrated in Fig. 4a–c in the main text.

An optimal modeling strategy keeps the model simple, yet biologically relevant and importantly capable of generating meaningful predictions. To this end, the models formulated are kept to minimal details, containing several biologically reasonable assumptions and simplifications: (1) The 1-site model is straightforward. Binding of the TF to its promoter follows mass-action kinetic laws of simple association/dissociation. We assume that the promoter becomes active and transcription is ON as soon as the binding site is occupied, and turned OFF when the TF dissociates from the promoter. (2) In the 2-site models, we assumed that binding between the TF and two sites occurs independently: the association/dissociate rates between the TF and either of the binding sites are the same when both sites are initially unoccupied, or when one of them is already occupied by the TF. In the OR gate model, transcription is ON as long as at least one site is occupied whereas in the AND gate model, transcription is ON only if both sites are occupied.

### Literature derived information on model parameters

NFκB p65 copies number has been measured in TNF-stimulated T-leukemia cell lines to be 120,000 [27]. Assuming the cell volume to be that of a typical hepatocyte, which is about 4 × 10^−12^ L [28], the copies number per cell is converted to concentration of about 50 nM. Thus, we used this number for the concentration of the TF in our models. Values of the association and dissociate rates of transcriptional factor binding to its DNA-biding site are rarely reported for mammalian systems; however, in bacteria such as *E. coli* these have been measured for the lac promoter and its TF regulators (repressor and activator) which is about 0.0027 nM^−1^ s^−1^ for association rate [29] and about 0.0023 s^−1^ for dissociation rate [30]. For our simulations, we used association rate = 0.002 nM^−1^ s^−1^ and dissociation rate = 0.002 s^−1^ in the 2-site models, and 0.02 s^−1^ for dissociation rates in the 1-site model as reasonable values.

### Model reactions and ODEs

#### 2-site ODE model

##### ODEs

$$\frac{{{\text{dTFP}}00\left( t \right)}}{{{\text{d}}t}} = - k_{{{\text{f}}1}} {\text{P}}00\left( {\text{t}} \right){\text{TF}}\left( t \right) - k_{{{\text{f}}3}} {\text{P}}00(t){\text{TF}}\left( t \right) - k_{{{\text{r}}2}} {\text{TFP}}10\left( t \right) + k_{{{\text{r}}3}} {\text{TFP}}01\left( t \right)$$

$$\frac{{d{\text{P}}00(t)}}{{{\text{d}}t}} = - k_{{{\text{f}}1}} {\text{P}}00\left( t \right){\text{TF}}\left( t \right) - k_{{{\text{f}}3}} {\text{P}}100\left( t \right){\text{TF}}(t) - k_{{{\text{r}}2}} {\text{TFP}}10\left( t \right) + k_{{{\text{r}}3}} {\text{TFP}}01$$

$$\frac{{{\text{dTF}}\left( t \right)}}{{{\text{d}}t}} = - k_{{{\text{f}}1}} {\text{P}}00\left( t \right){\text{TF}}\left( t \right) - k_{{{\text{f}}2}} {\text{TF}}\left( t \right){\text{TFP}}10\left( t \right) - k_{{{\text{f}}3}} {\text{P}}00\left( t \right){\text{TF}}\left( t \right) - k_{{{\text{f}}4}} {\text{TF}}\left( t \right){\text{TFP}}01\left( t \right) + k_{{{\text{r}}1}} {\text{TFP}}10\left( t \right) + k_{{{\text{r}}2}} {\text{TFP}}11\left( t \right) + k_{{{\text{r}}3}} {\text{TFP}}01\left( t \right) + k_{{{\text{r}}4}} {\text{TFP}}11\left( t \right)$$

$$\frac{{{\text{dTFP}}01\left( t \right)}}{{{\text{d}}t}} = k_{{{\text{f}}3}} {\text{P}}00\left( t \right){\text{TF}}\left( t \right) - k_{{{\text{f}}4}} {\text{TF}}\left( t \right){\text{TFP}}01\left( t \right) - k_{{{\text{r}}3}} {\text{TFP}}01\left( t \right) + k_{{{\text{f}}4}} {\text{TFP}}11\left( t \right)$$

$$\frac{{{\text{dTFP}}10\left( t \right)}}{{{\text{d}}t}} = k_{{{\text{f}}1}} {\text{P}}00\left( t \right){\text{TF}}\left( t \right) - k_{{{\text{f}}2}} {\text{TF}}\left( t \right){\text{TFP}}10\left( t \right) - k_{{{\text{r}}1}} {\text{TFP}}10\left( t \right) + k_{{{\text{r}}2}} {\text{TFP}}11\left( t \right)$$

$$\frac{{{\text{dTF}} - {\text{P}}11(t)}}{{{\text{d}}t}} = k_{{{\text{f}}2}} {\text{TFP}}10\left( t \right) + k_{{{\text{f}}4}} {\text{TF}}\left( t \right){\text{TFP}}01\left( t \right) - k_{{{\text{r}}2}} {\text{P}}11\left( t \right) - k_{{{\text{r}}4}} {\text{TFP}}11(t)$$

#### 1-site ODE model

##### Reactions

$${\text{TF}} + {\text{P}}0 \mathop{\rightleftarrows}_{{k_{\rm f1}}}^{{k_{\rm r1}}} {\text{TFP}}1$$

##### ODEs

$$\frac{{{\text{dP}}0\left( t \right)}}{{{\text{d}}t}} = k_{{{\text{r}}1}} {\text{TFP}}1\left( t \right) - k_{{{\text{f}}1}} {\text{P}}0\left( t \right){\text{TF}}\left( t \right)$$

$$\frac{{{\text{dTF}}\left( t \right)}}{{{\text{d}}t}} = k_{{{\text{r}}1}} {\text{TFP}}1\left( t \right) - k_{{{\text{f}}1}} {\text{P}}0\left( t \right){\text{TF}}\left( t \right)$$

$$\frac{{{\text{dTFP}}1\left( t \right)}}{{{\text{d}}t}} = k_{{{\text{f}}1}} {\text{P}}0\left( t \right){\text{TF}}\left( t \right) - k_{{{\text{r}}1}} {\text{TFP}}1\left( t \right)$$

The same parameter values as given in Table 1 are used for the 1-site model.

**Table 1:** Reactions and parameter values of the 2-site models

Reactions	Parameter values
${\text{TF}} + {\text{P}}00 \mathop{\rightleftarrows}\limits_{{k_{\rm r1}}}^{{k_{\rm f1}}} {\text{TFP}}10$	*k*_f1_ = 0.002 *k*_r1_ = 0.002
${\text{TF}} + {\text{P}}10 \mathop{\rightleftarrows}\limits_{{k_{\rm r2}}}^{{k_{\rm f2}}} {\text{TFP}}11$	*k*_f2_ = 0.002 *k*_r2_ = 0.002
${\text{TF}} + {\text{P}}00 \mathop{\rightleftarrows}\limits_{{k_{\rm r3}}}^{{k_{\rm f3}}} {\text{TFP}}01$	*k*_f3_ = 0.002 *k*_r3_ = 0.002
${\text{TF}} + {\text{P}}01 \mathop{\rightleftarrows}\limits_{{k_{\rm r4}}}^{{k_{\rm f4}}} {\text{TFP}}11$	*k*_f4_ = 0.002 *k*_r4_ = 0.002

The association and dissociation kinetic rates (*k*) used have units of nM^−1^ s^−1^ and s^−1^, and all concentration of all molecular species have unit of nM. TF refers to transcription factor. P refers to the promoter, with 00 referring to bound TF on both binding sites, 01 and 10 referring to occupancy of either the first or the second binding site and 11 referring to occupancy of both binding sites. Initial conditions: TF(0) = 10, P0(0) = 50, all remaining species are zero

### Hill function fitting and Hill coefficient calculation

The steepness of a dose–response curve is often indicated by the Hill coefficient value resulting from fitting the curve with a Hill function [31]. The following Hill function was used for fitting dose–response data curves (Supplementary Figure S3):

$${\text{Hill function}} = \beta + R_{\hbox{max} } \frac{{x^{\text{H}} }}{{x_{50}^{\text{H}} + x^{\text{H}} }}$$

where *β* is the offset level, *R*_max_ is the maximal response, *x*_50_ is the half-maximal threshold and *H* is the Hill coefficient which indicates strength of the switch. Fitting was implemented with the NonlinearModelFit function in Mathematica 8.

### Sequence alignment

Sequences and species alignment for the COX2 promoters were obtained from the University of California Santa Cruz Genome Browser [32].

### Reagent

Tumor necrosis factor α (TNFα) was obtained from Sigma (Ireland).

### Statistical analysis

All experiments were carried out a minimum of *n* = 3 independent times unless otherwise indicated and data are expressed as the mean ± SEM. Statistical significance was calculated by Student’s *t* test for the comparison of two datasets or one-way ANOVA followed by Bonferroni’s multiple comparison test (Microcal Origin Lab 7.5; Origin Lab) for more than two datasets. *P* < 0.05 was considered statistically significant.

## Results

### Species differences between human and mouse COX2 protein expression

**Fig. 1:**
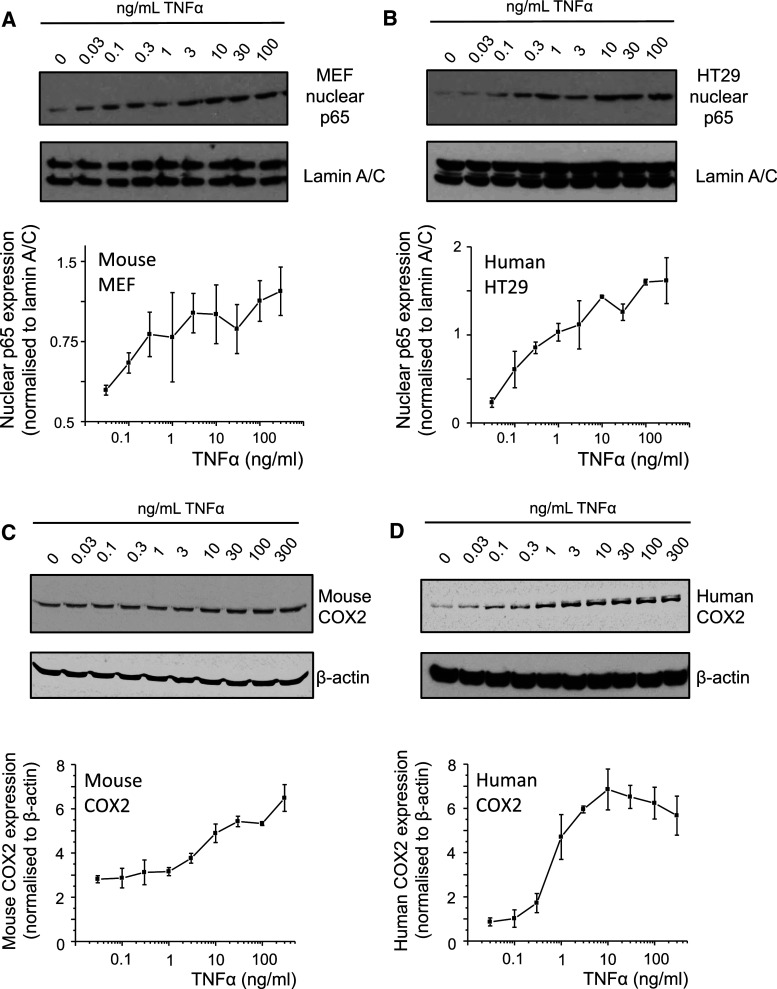
TNFα-induced nuclear localization of p65 and COX2 protein expression in human and mouse. **a**, **b** Nuclear p65 in human HT29 and mouse MEF cells stimulated with TNFα (0–100 ng/ml) for 1 h (*n* = 3, data shown as normalized to lamin A/C). **c**, **d** Expression of COX2 protein in human HT29 and mouse MEF cells stimulated with TNFα (0–100 ng/ml) for 6 h (*n* = 3, data shown as normalized to β-actin)

The inflammatory stimulus TNFα induces nuclear localization of p65, one of the main subunits of NFκB [17, 18]). We indeed observe that this response is linear in both mouse MEF cells and human HT29 cells exposed to increasing concentration of TNFα (Fig. 1a, b). The nuclear localization of p65 resulted in a proportional increase in COX2 protein expression in mouse cells, but not in the human cells (Fig. 1c, d; Supplementary Figures S3A and B), suggesting a species difference in COX2 expression in response to TNFα downstream of p65 nuclear translocation. Thus, we investigated the promoter of COX2 to identify possible differences in *cis*-regulatory response elements.

### Analysis of the human COX2 promoter reveals an additional NFκB response element

**Fig. 2:**
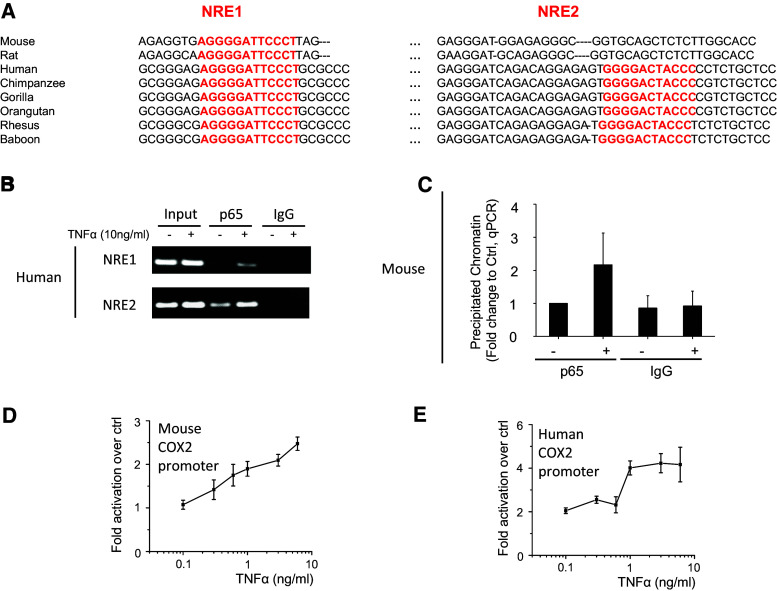
Analysis of the human and mouse COX2 promoters. **a** Cross-species sequence alignment of the COX2 promoters shows that the NRE1 is conserved among species analyzed, but the NRE2 (in *red*) is only observed among primates (including human) and absent in rodents (including mouse). **b** Chromatin immunoprecipitation analysis shows binding of p65 to both NREs on the hCOX2 promoter in HEK293 cells stimulated with TNFα (10 ng/ml). The products of the PCR reactions where run on an agarose gel and a representative picture from four independent experiments are shown. **c** Chromatin immunoprecipitation analysis shows binding of p65 to the NRE on the mCOX2 promoter in MEF cells stimulated with TNFα (10 ng/ml) (*n* = 3). Quantification of p65 binding to the mouse NRE was performed using the Taqman system for qPCR, results are shown as fold change to the p65-immunoprecipitation of unstimulated cells after normalization to input controls. TNFα-induced luciferase activity under the control of mouse (**d**) and human (**e**) COX2 promoters expressed in HEK293 cells (shown as fold activation over unstimulated; *n* = 4)

It has been shown that a single nucleotide can determine which co-factors are recruited to the NFκB response element (NRE) [33], and speculated that species-specific differences in the COX2 production between mice and human could be caused by differences in the NRE element. The mouse COX2 (mCOX2) promoter was previously shown to contain only 1 functional NFκB response element (NRE; [16]), which is conserved among species (termed NRE1; Fig. 2a). Thus, we considered the hypothesis of different co-factor recruitment unlikely. Interestingly, alignment of the COX2 promoters for different species reveals that the second NRE (termed NRE2) present in humans is conserved only among primates but not in other species such as rodents (Fig. 2a). Chromatin immunoprecipitation experiments in human HEK293 and mouse MEF cells show that there is increased p65 bound to the two human NRE sites (Fig. 2b) and to the single mouse NRE site (Fig. 2c) upon stimulation with tumor necrosis factor alpha (TNFα). Therefore, we decided to determine whether the presence of the second, active, NRE element in the human promoter could be responsible for the species-specific differences in COX2 regulation. We reason that the function of the two NREs in the human COX2 promoters can be revealed by examining the response of the promoters to increasing concentration of TNFα.

We next compare the transcriptional activities of the human COX2 promoter to the mouse equivalent (details of the sequence are shown in Supplementary Figures S1–S2). These experiments are performed in HEK293 cells, so that the responses of the human and mouse promoters to the same inflammatory stimulus can be compared. Our data indicate that the response from the mouse COX2 promoter is linear (Fig. 2d; Supplementary Figure S3C), whereas the response from the human COX2 is switch-like (Fig. 2e; Supplementary Figure S3D). Taken together, the pattern of the protein expression shows good correlation with the promoter activity (Fig. 1), indicating that the NFκB-regulated promoter has a strong influence on the COX2 protein expression.

### An AND gate regulating the human cyclooxygenase 2 expression

**Fig. 3:**
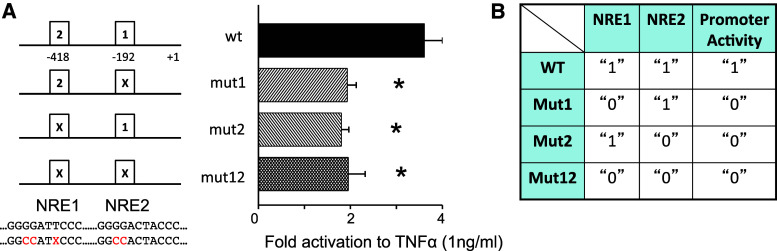
The two NFκB response elements of COX2 form a functional logic AND gate. **a** Transcriptional activity of hCOX2 wild-type promoter or mutants in response to TNFα (shown as fold activation over unstimulated; *n* = 4). **b** Truth table showing the relationship between promoter activity (1 = active due to NFκB, 0 = basal) and presence (1) or absence (0) of NRE for wild-type and mutants hCOX2 promoter. Significant differences (*p* < 0.05) are denoted by *asterisk*

To determine the contribution of each NRE to hCOX2 expression, we have generated mutants of the NREs of the hCOX2 promoter and find that the promoter activities resulting from either mutation of single sites or dual mutations in both sites are similarly significantly lower than the activity of the wild type in response to TNFα (Fig. 3a). These activity responses can be represented on a truth table (Fig. 3b) which displays how the output (1 for promoter activity “ON” and 0 for promoter activity “OFF”) relates to various combinations of the inputs (1 for “presence” and 0 for “absence” of the response elements). The table shows that promoter activity is observed only when both NREs are intact. Consequently, the regulation of the hCOX2 promoter is consistent with the functioning of an AND gate. In the general case, the output of an AND gate assumes two distinct states that may differ in meaning (like “on” and “off”) or correspond to two different values on a discrete (like “0” and “1”) or continuous (like −5 and +5 V) scale. Moreover, the AND gate exhibits two input ports and one of the two output signals can only be observed when both input ports are activated. In all other cases, the other output signal can be observed. In our context, the two output states correspond to low and high levels of promoter activity, while the two input ports correspond to the two NREs. Finally, note that also at a higher concentration of TNFα (Supplementary Figure S4) the promoter response is still consistent with the functioning of an AND gate.

### Mathematical models of NREs-regulated transcriptional activity

**Fig. 4:**
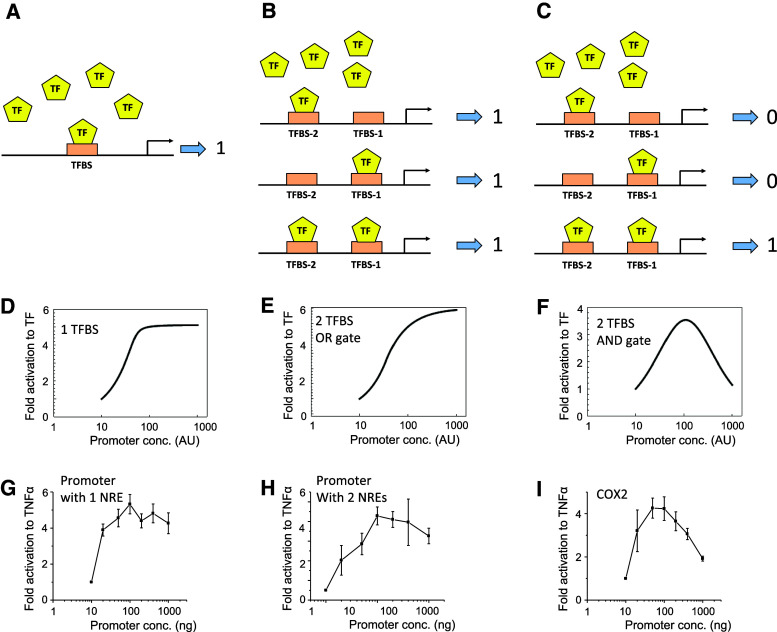
Mathematical models of promoter activity. Simplified schemes showing binding of TFs on promoters with 1 transcription binding site (TFBS) (**a**), or 2 TFBS arranged as OR (**b**) or AND gates (**c**). Promoter is either activated (1) or not (0). **d**–**f** Mathematical predictions of the transcriptional activity for each scheme, at a fixed concentration of TF but varying concentration of promoters. Model association/dissociation rates used for plotting panel (**f**) are 0.001 and 0.2, respectively. Values for other *panels* are given in “Materials and methods”. **g**–**i** Experimental validation of predictions using artificial promoters or the hCOX2 promoter expressed in HEK293 cells in response to TNFα (1 ng/ml). Data are shown as fold activation over unstimulated (*n* = 4–5)

To explore the functional and dynamic property of our proposed logic AND gate, we constructed a set of quantitative kinetic models of promoter activity based on the binding of a transcription factor (TF) to a promoter containing either one binding site (TFBS, Fig. 4a), two sites with an OR gate (Fig. 4b) or two sites joined as an AND gate (Fig. 4c). Details of the model development including model equations and parameter values are given in the “Materials and methods” section. Model simulations predict that, at a given concentration of the input TF, the promoter activity increases following a monotonic, saturating pattern in response to increasing concentration of the promoter in the 1-site or 2-site OR gate models (Fig. 4d, e). However, for the 2-site AND gate model, as in the case of our hCOX2 expression system, the transcriptional activity is predicted to follow a distinct bell-shaped, biphasic dependence on the promoter concentration (Fig. 4f). Increasing the promoter concentration enhances the promoter activity at lower concentrations, while a further increase beyond an optimal level instead attenuates the activity. Mechanistically, a very high concentration of the promoter will sequester individual molecules of the TF to either of its binding sites forming TF-promoter complexes that are incomplete, i.e., having just site 1 or site 2 occupied by the TF (no transcriptional activity), but only few complexes with both sites occupied (transcriptionally active). This effect is generally known as the prozone effect [34], which has been previously reported for scaffold proteins [35].

To further show that the predicted bell-shaped feature is a robust property of the 2-site AND gate model, we carried out unbiased simulations where the model kinetic rate constants were allowed to randomly vary within wide ranges of physiologically plausible values. For 1,000 random parameter sets simulated, the 2-site OR gate model only showed monotonic, saturating dose–response curves for the promoter activity across simulated sets (Supplementary Figures S7A). On the contrary, the same analysis for the 2-site AND gate model consistently displayed the bell-shaped activity dependence across the sets despite having variability in the activity amplitude and optimal peak (Supplementary Figures S8C). Calculating the mean curve and curves within one standard deviation further confirmed the robustness of the predicted bell-shape response (Supplementary Figs. 7B vs. 7C). Taken together, these ensemble simulations demonstrate that the prozone effect is an exclusive and robust feature of the 2-site AND gate model.

To experimentally test the predicted prozone effect for various expression systems, we use a sequence from the interleukin-6 promoter which was previously shown to contain only one functional NRE [24] as example of the 1-site model (Fig. 4g, detail of the sequence used is shown in Supplementary Material). We next duplicate this sequence to create a promoter with two binding sites for NFκB (Fig. 4h; detail of the sequence used is shown in Supplementary Material), exemplifying the 2-site model. Using a constant stimulus level of TNFα and transfecting increasing amount of plasmid DNA encoding the different promoter constructs, promoter activity responses measured for the three cases agree well with model predictions, with a biphasic pattern observed only for the AND gate hCOX2 promoter (Fig. 4g–i). Thus, we show, using mathematical modeling and experimental validation, that the two NREs on the hCOX2 promoter form a functional AND gate which requires both NREs to be occupied by NFκB to be active.

### Mathematical prediction of NREs-regulated COX2 activity in mouse and human

**Fig. 5:**
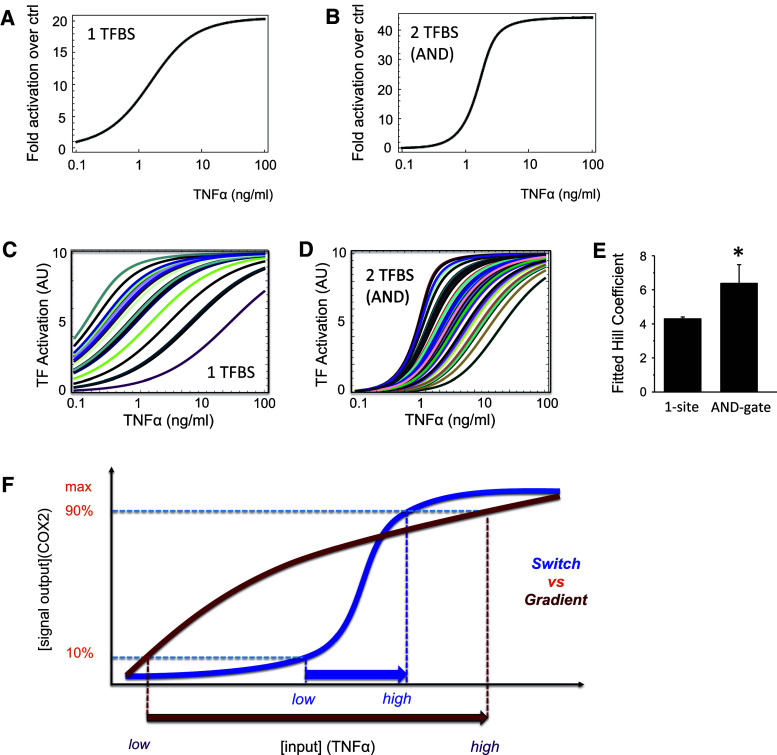
Digital noise filtering property of the AND logic gate in the hCOX2 promoter. **a**, **b** Mathematical predictions of the transcriptional activity of the promoter with TFBS site (corresponding to mouse COX2) or 2 TFBS arranged as an AND gate (corresponding to human COX2). **c**–**e** Ensemble simulation of the 1-site model vs. 2-site AND gate model. **c** Simulations of the 1-TFBS model for 100 random parameter sets randomly drawn from the ranges (0.0001, 0.01) and (0.001, 0.1) for association and dissociation kinetic rates, respectively. Parameter units are given in Table 1. **d** Similar simulations as in **c** for the 2-site AND gate model. **e** Comparison of level of switchness of the dose–response curves (**c**, **d**) quantified as the fitted Hill coefficient for 1-site model vs. 2-site AND gate model. Significant differences (*p* < 0.05) are denoted by *asterisk*. **f** Comparison between a graded or switch-like response: for a similar increase in output (from 10 to 90 %), a graded response (*red line*) requires a larger change in input than for a switch-like response (*blue line*)

Our mathematical model predicts that a promoter with a single TFBS is likely to produce a linear increase in promoter activity (Fig. 5a), whereas one with two sites that function like an AND gate will instead produce a sigmoidal, switch-like response (Fig. 5b). To test if this prediction is robust and that the sigmoidal response is a more characteristic property of the 2-site AND gate model compared to the 1-site model, we carried out similar ensemble simulations with large number of randomized parameter sets as described in the previous section. We varied the kinetic rate constants in both models over wide ranges of values within typical physiological intervals and for each parameter set, we quantified the switchness of the dose–response curve by calculating the corresponding Hill coefficient (Fig. 5c–e). The 2-site AND gate model generally displays a much more switch-like activity response compared to that of the 1-site model. This is further corroborated by the quantified Hill coefficient, which is statistically significantly higher for the 2-site AND gate than the 1-site model (Fig. 5e). Taken together, the model predictions for the COX2 promoter activity show good correlation with the experimental observed promoter activity (Fig. 2c, d), indicating that the human COX2 is likely to be regulated by the NFκB-regulated AND gate in its promoter.

When there is a graded signal output to an input, the cell requires a large change in the inflammatory stimulus (input) to achieve a maximum expression of COX2 (signal output; Fig. 5f, red line). However, if the output/input curve is switch-like, maximal COX2 is rapidly expressed in response to a much smaller change in the inflammatory stimulus (Fig. 5f, blue line). Furthermore, the switch-like behavior provides a minimum threshold for activation at which the inflammatory stimulus needs to reach before COX2 can be maximally expressed.

## Discussion

Cyclooxygenase 2, a key regulatory enzyme of the prostaglandin/eicosanoid pathway, is highly induced by pro-inflammatory cytokines in an NFκB-dependent manner. Here, we show that the presence of the NFκB-regulated AND gate in the human COX2 promoter acts as a noise filter such that a threshold of NFκB activation is required before the promoter becomes active and initiates transcription. While the AND gate uncovered for the human COX2 promoter is composed of two response elements for NFκB, it is likely that other AND gates may exist with multiple response elements for either the same transcription factor or a combination of two or more different transcription factors [33]. For example, there is an indication of similar regulatory mechanisms from the promoter assays for the NFκB regulation of Receptor for Advanced Glycation Endproducts (RAGE [36]) and C-X-C motif chemokine 10 (CXCL10 [37]).

Boolean logic elements such as AND and OR gates have been identified both at the level of cell signaling [38, 39] and the genetic level [26, 40–42]. In these studies, typically two different input signals have been considered representing either different pathways or different transcription factors. In contrast, we have developed our arguments in analogy to electronic Boolean gates that feature spatially separated input ports that receive the same kind of signals. The two NREs are considered as two input ports at separated locations that respond to the same kind of input in terms of NFκB activation. Binding of the NFκB transcriptional factor to only one of the two sites is analogous to an electronic AND gate receiving a signal at only one of its input ports. In this case, the COX2 expression system, as well as the electronic case, responds as if no signal was received at all and delivers a low, basal response. If bindings to both NREs occur, then this scenario is comparable with an electronic AND gate that receives signals at both input ports. In the COX2 as well as the electronic gate, the regulatory machinery responds with an output signal that is qualitatively and significantly higher than the basal response. In other words, we may say that our observations suggest a strong cooperative effect between the two NRE sites. The cooperativity effect exists in the sense that the two sites require one another to respond appropriately to NFκB activation. While we have shown that the two NREs of the human COX2 promoter confer an AND gate-like behavior to the transcriptional response, we are aware that a full explanation of how this behavior is achieved will require further investigations, such as detailed analysis of the sequence and nucleic acid tertiary structure [43].

TNFα induces a similar linear nuclear localization of p65 in both human HT229 and mouse MEF cells, but only the human cell displayed a switch-like expression of COX2 protein. We thus propose that the absence of the NFκB-regulated AND gate in the mouse COX2 promoter is most likely responsible for its linear response in promoter activity and protein expression to TNFα. Our data show that the mouse COX2 is sensitive to a spectrum of TNFα concentration, whereas the human COX2 has a narrower range. The regulation of COX2 is complex and very likely involves crosstalk with other transcription factors [8–11], which may be affected by TNFα. However, in this paper, we concentrate on TNFα-induced NFκB regulation of COX2 only.

Our experimental evidence comes from measuring transcriptional activity and protein expression as a population of cells was exposed to TNFα. This measurement is the average of the total population and we have assumed that all the cells have responded to a similar fashion. However, it is possible that the average measurement is primarily due to a fraction of the cells in the population which have responded, with the remaining unresponsive or less responsive. Examining this possibility requires single cell measurement for protein expression and potentially a surrogate marker for transcriptional activity [44], which is an area for future investigation.

Species-specific differential regulatory mechanisms of gene expression, as demonstrated here for COX2, may contribute to the recently reported discordance observed between human and mouse models of inflammatory diseases [7]. The study of these mechanisms will help us to understand when the findings from biomedical research in mouse models are relevant to human disease [45]. Several examples of these mechanisms exist. For example, the transcription factor ETS1 is responsible for the mouse specific expression of the T cell factor Thy-1 in the thymus, and its preferential recruitment to the proximal promoter of human genes but not mice genes has been suggested to contribute to the immune system differences between mice and human [45]. Different patterns of histone methylation and acetylation have also been found to be associated to the species-specific expression of genes across several representative tissues, indicating that epigenetic regulation is also a key mechanism leading to differential gene expression in mice and human tissues [46]. In addition to the differential binding of TF to the promoter and epigenetics marks, differences in the *cis*-regulatory elements (CRE, TF binding sites and associated sequences required for transcription) can also contribute to species-specific expression patterns of the same gene [47]. Species-specific differences in gene expression can arise due to novel CRE, but also through mutations in pre-existing CRE including insertions, deletions, duplications and changes in the DNA strand of the TF binding site [47]. Our work here identifies the presence of an AND gate-like behavior composed of 2 NRE sites in the human COX2 promoter, that is absent in the mouse promoter, as a novel regulatory mechanisms leading to species-specific divergence in the expression of this key inflammatory gene.

Candidate drugs are usually developed and tested in mouse models, but have very poor success rates when moved to clinical trials [7]. COX2 inhibitors have been withdrawn due to major adverse effects [6]. Our results suggest that the induction of COX2 in human is threshold controlled, i.e., in the absence of sufficient inflammatory stimulus, COX2 is not induced. It is possible in these circumstances that COX2 inhibitors instead target other pathways non-specifically, potentially leading to the reported adverse effects. Thus, we propose that the development of future COX2 inhibitors should take into account the threshold-controlled regulation of COX2 expression by inflammatory stimulus and species-specific differences.

In conclusion, the logic AND gate comprised of the two NREs characterized in this study represents a novel regulatory mechanism for the multitude regulation modes exhibited by NFκB [18, 48]. Our data indicate that this AND gate acts as a noise filter to tightly regulate the expression of COX2. It is very likely that similar AND gates may exist on other genes to regulate their transcription [33, 36, 37] and our study provides the foundation for further studies on understanding the regulation of genes with switch-like expression [49–51]. Furthermore, we show that COX2 expression is differentially regulated in human and mouse, and attribute this difference to the presence of the AND gate in its human promoter but absence in the mouse homolog. These findings may explain the variance between animal models of inflammatory diseases and differences to human conditions and highlight the need for careful selection of appropriate substitute models to represent human inflammatory diseases.

### Electronic supplementary material

Below is the link to the electronic supplementary material.

Supplementary material 1 (PDF 2368 kb)

## Abbreviations

COX1: Cyclooxygenase 1
COX2: Cyclooxygenase 2
hCOX2: Human cyclooxygenase 2
mCOX2: Mouse cyclooxygenase 2
TNFα: Tumor necrosis factor alpha
NFκB: Nuclear factor kappa B
NRE: NFκB response element
Gluc: Gaussia luciferase
pGluc: Plasmid encoding Gluc
CRE: *Cis*-regulatory elements
