# Supplementary material for: Species differential regulation of COX2 can be described by an NFκB-dependent logic AND gate
Source: Cell Mol Life Sci. 2015 Feb 20;72(12):2431–43. doi: 10.1007/s00018-015-1850-1 (PMC4439527; doi:10.1007/s00018-015-1850-1)
Supplement: Supplementary file 1 — Supplementary material 1 (PDF 2368 kb) [file 18_2015_1850_MOESM1_ESM.pdf]

Species differential regulation of COX2 can be described by an NFkB-dependent logic AND gate

Lan K Nguyen[1, *], Miguel A S Cavadas[1,2 *], Boris N Kholodenko[1], Till D Frank[3] and Alex Cheong[1, 4]

[1]Systems Biology Ireland, University College Dublin, Dublin 4, Ireland.
[2]Instituto Gulbenkian de Ciencia, Oeiras, Portugal.
[3]Center for the Ecological Study of Perception and Action, University of Connecticut, Storrs, CT 06269, USA.
[4]School of Life and Health Sciences, Aston University, Birmingham, B4 7ET, UK.

[*] These authors contributed equally to this work.

Supplementary Material

TCTGAGCAGCGAGCACGTCAGACTGCGCCCCAGTGGGGAGAGGTGAGGGGATTCCCTTAGTT
AGGACCTTAGATCCCGGGAGGGGAAGCTGTGACACTCTTGAGCTTTTAGGCCCCCACTGGAT
GCGCGACTGGGAGGAAACCGGAGACCCCAAAGAGCGCCAGACTAGGCGCAGACTCAGCGAAC
CACAGGGCGCCTGGAGGGATGGAGAGGGCGGTGCAGCTCTCTTGGCACCACCTGGGGCAGCC
AAGGGCAGCTTCCCGGCTTCCTTCGTCTCTCATTTGCGTGGGTAAAAGCCTGCCGCTGCGGT
TCTT

Supplementary Figure S1. Sequence of the mouse COX2 promoter used to create pGluc-mCOX2. The NFκB response element is highlighted in yellow.

GCTATTTCATTCCACAAAATAAGAGTTTTTTAAAAAGCTATGTATGTATGTGCTGCATATAG
AGCAGATATACAGCCTATTAAGCGTCGTCACTAAAACATAAAACATGTCAGCCTTTCTTAAC
CTTACTCGCCCCAGTCTGTCCCGACGTGACTTCCTCGACCCTCTAAAGACGTACAGACCAGA
CACGGCGGCGGCGGCGGGAGAGGGGATTCCCTGCGCCCCCGGACCTCAGGGCCGCTCAGATT
CCTGGAGAGGAAGCCAAgTGTCCTTCTGCCCTCCCCCGGTATCCCATCCAAGGCGATCAGTC
CAGAACTGGCTCTCGGAAGCGCTCGGGCAAAGACTGCGAAGAAGAAAAGACATCTGGCGGAA
ACCTGTGCGCCTGGGGCGGTGGAACTCGGGGAGGAGAGGGAGGGATCAGACAGGAGAGTGGG
GACTACCCCCTCTGCTCCCAAATTGGGGCAGCTTCCTGGGTTTCCGATTTTCTCATTTCCGT
GGGTAAAAAACCCTGCCCCCACCGGGCTTACGCAATTTTTTTAAGGGGAGAGGAGGGAAAAA
TTTGTGGGGGGTACGAAAAGGCGGAAAGAAACAGTCATTTCGTCACATGGGCTTGGTTTTCA
GTCTTATAAAAAGGAAGGTTCTCTCGGTTAG

Supplementary Figure S2. Sequence of the human COX2 promoter used to create pGluc-COX2. The NFκB response elements are highlighted in yellow.

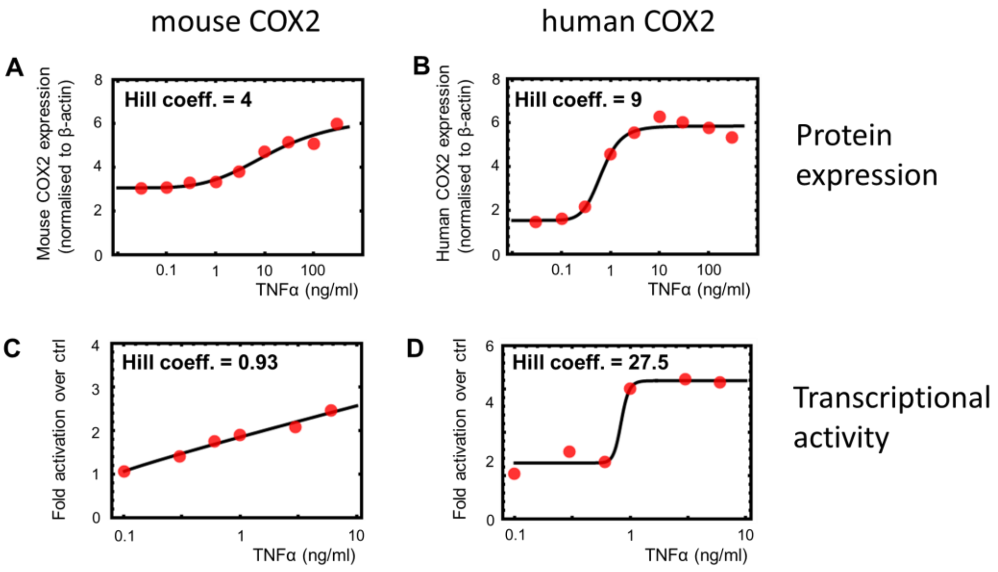

Supplementary Figure S3. Analysis of protein expression and transcriptional activity as a function of TNFα concentration. (A, B) The data on COX2 protein expression from mouse (A) and human (B) cells (from Figures 1C and D) were fitted with the Hill equation. **(C, D)** The data on transcriptional activity from mouse (C) and human (D) COX2 promoter (from Figures 2C and D) were fitted with the Hill equation. Hill coefficients are given in the respective graphs.

A

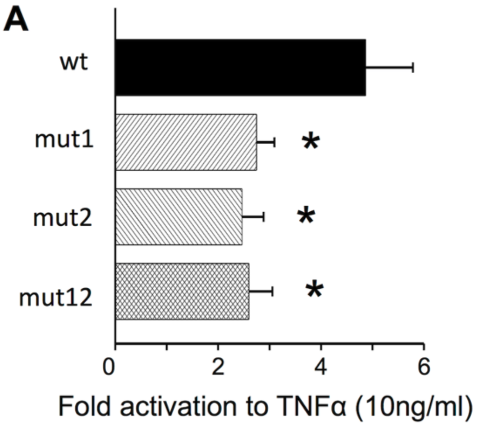

B

	NRE1	NRE2	Promoter Activity
**WT**	“1”	“1”	“1”
**Mut1**	“0”	“1”	“0”
**Mut2**	“1”	“0”	“0”
**Mut12**	“0”	“0”	“0”

Supplementary Figure S4. The AND gate is functional at higher concentration of TNFα. (A) Transcriptional activity of hCOX2 wild-type promoter or mutants in response to 10ng/ml of TNFα (shown as fold activation over unstimulated; $n = 4$). **(B)** Truth table showing the relationship between promoter activity (1 = active due to NFκB, 0 = basal) and presence (1) or absence (0) of NRE for wild-type and mutants hCOX2 promoter. Significant differences ($p<0.05$) are denoted by *.

CTAGCCTCAATGACGACCTAAGCTGCACTTTTCCCCCTAGTTGTGTCTTGCcATGCTAAAGG
ACGTCACATTGCACAATCTTAATAAGGTTTCCAATCAGCCCCACCCGCTCTGGCCCCACCCT
CACCCTCCAACAAAGATTTATCAAATGTGGGATTTTCCCATGAGTCTCAATATTAGAGTCTC
AACCCCCAATAAATATAGGACTGGAGATGTCTGAGGCTCATTCTGCCCTCGAGCCCACCGGG
AACGAAAGAGAAG

Supplementary Figure S5. Sequence of the human IL6 promoter used for creating the pGluc-IL6. The NFκB response element is highlighted in yellow.

```
CTAGCCTCAATGACGACCTAAGCTGCACTTTTCCCCCTAGTTGTGTCTTGCcATGCTAAAGG
ACGTCACATTGCACAATCTTAATAAGGTTTCCAATCAGCCCCACCCGCTCTGGCCCCACCCT
CACCCTCCAACAAAGATTTATCAAATGTGGGATTTTCCCATGAGTCTCAATATTAGAGTCTC
AACCCCCAATAAATATAGGACTGGAGATGTCTGAGGCTCATTCTGCCCTCGAGCCCACCGGG
AACGAAAGAGAAGTCTGCCCTCGAGCCCACCGGGAACGAAAGAGAAGGAATTCCTAGCCTCA
ATGACGACCTAAGCTGCACTTTTCCCCCTAGTTGTGTCTTGCcATGCTAAAGGACGTCACAT
TGCACAATCTTAATAAGGTTTCCAATCAGCCCCACCCGCTCTGGCCCCACCCTCACCCTCCA
ACAAAGATTTATCAAATGTGGGATTTTCCCATGAGTCTCAATATTAGAGTCTCAACCCCCAA
TAAATATAGGACTGGAGATGTCTGAGGCTCATTCTGCCCTCGAGCCCACCGGGAACGAAAGA
GAAG
```

Supplementary Figure S6. Sequence used for creating the pGluc-2xIL6. The NFκB response elements are highlighted in yellow. The green highlighted sequence refers to the original pGluc vector.

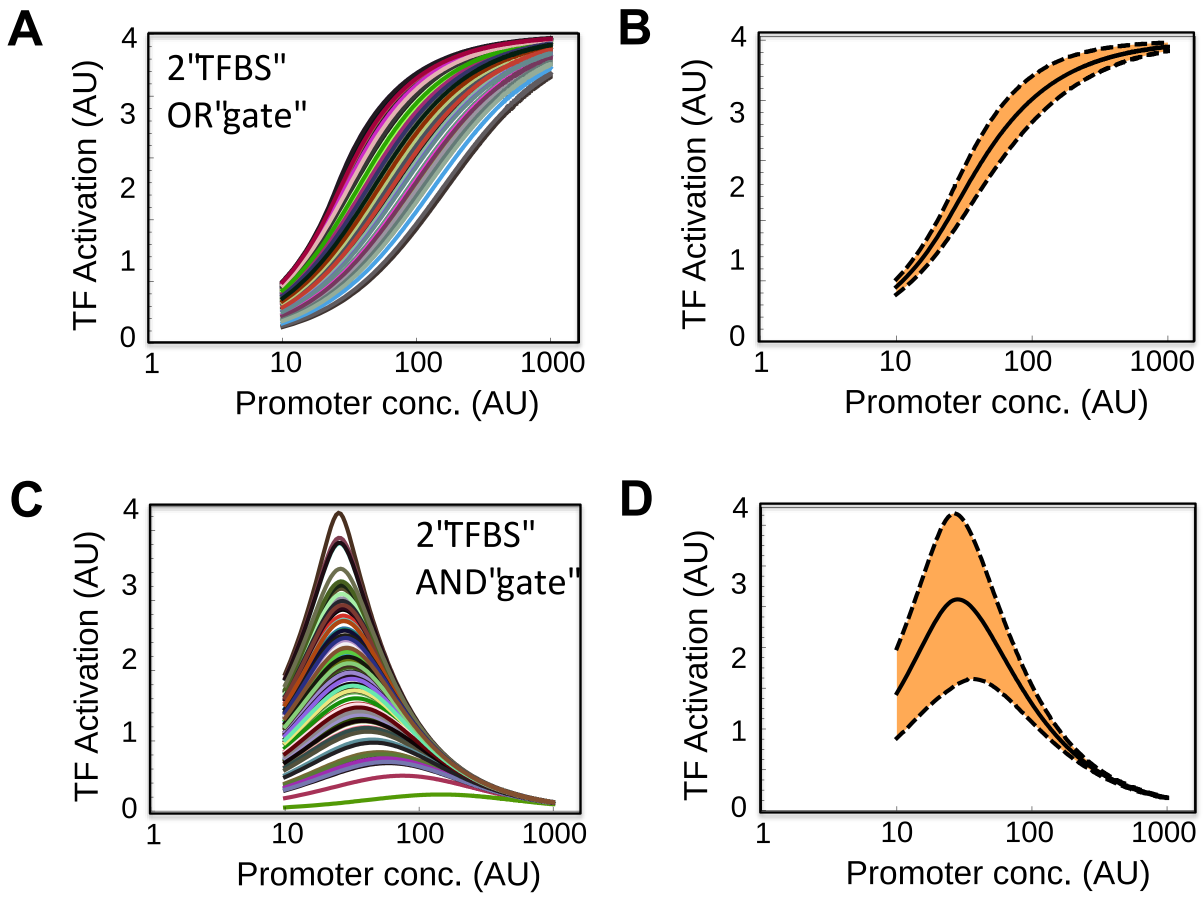

Supplementary Figure S7. Ensemble simulation of the 2-sites AND- vs OR-gate model. (**A**) Simulations of the OR-gate model for 1000 random parameter sets randomly drawn from the ranges [0.0001,0.01] and [0.001,0.1] for association and dissociation kinetic rates, respectively. Parameter units are given in Table 1. (**B**) Mean (solid) and +/- one standard deviation (dashed) curves calculated from the dose-response curves in panel (**A**). (**C, D**) Similar simulations as in (**A, B**) for the AND-gate model.
